# Consumer identity but not food availability affects carabid diet in cereal crops

**DOI:** 10.1007/s10340-023-01620-w

**Published:** 2023-04-24

**Authors:** Yasemin Guenay-Greunke, Harald Trager, David A. Bohan, Michael Traugott, Corinna Wallinger

**Affiliations:** 1 Applied Animal Ecology, Department of Zoology, University of Innsbruck, Technikerstraße 25, 6020 Innsbruck, Austria; 2 Institute of Interdisciplinary Mountain Research, IGF, Austrian Academy of Sciences, Technikerstraße 21a, 6020 Innsbruck, Austria; 3 Agroécologie, AgroSup Dijon, INRAE, Université Bourgogne Franche-Comté, 21000 Dijon, France

**Keywords:** Carabidae, Metabarcoding, Seed predation, Trophic interactions, Slugs, Pest control

## Abstract

Understanding trophic interactions in agroecosystems is crucial for harnessing ecosystem services such as pest control, thus enabling a reduction in pesticide use. Carabid beetles (Coleoptera: Carabidae) have the potential to regulate not only insect pests but also weed seeds and slugs. The aim of this study was to investigate the food choice of different carabid species in the experimental setting of a cereal field with varying seed and slug prey availability during the season. In addition to varying food availability, the effects of species identity and season on carabid food choice should also be closely examined. Therefore, the gut contents of 1,120 beetles of eight carabid species were screened for the DNA of plants, aphids, springtails, earthworms and slugs via diagnostic multiplex PCR and a nested metabarcoding approach for plant species identification. Plant DNA was detected far more often (72%) than the various animal prey types (less than 12.5% each). Within the plant detections, 80 weed species were identified in the metabarcoding, with *Galinsoga parviflora/quadriradiata* (*Galinsoga* spp.—quickweeds) as the most frequently detected species. Carabid food choice was driven by their species identity and seasonality, while no effect of increased availability of seeds and slugs on their food choice was detected. While weed seeds seem to be an important food source for carabids, their availability does not directly affect the carabid diet. The importance of consumer identity and seasonality highlight the need for a diverse carabid species community for resilient pest control services.

**Supplementary Information:**

The online version contains supplementary material available at 10.1007/s10340-023-01620-w.

## Key message

- Carabid beetles have the ability to regulate both invertebrate pests and weed seeds.
- However, the trophic guilds among these beneficial organisms remain uncertain.
- It is also unclear whether and to what extent their ecosystem services vary during the season.
- Using molecular gut content analyses, weed seed DNA was most frequently detected in their samples.
- Diet choice was found to be influenced by carabid species and time, but not on seed availability.

## Introduction

Carabid beetles (Coleoptera: Carabidae) provide ecosystem services such as weed seed predation (Honek et al. 2003; Kulkarni et al. 2015; Tooley and Brust 2002) and pest regulation, including the predation on slugs (Bohan et al. 2000; Symondson et al. 2002a, 2002b), by their feeding behaviour. To use these ecosystem services sustainably, further insights into the complex networks of these beneficial insects and their various prey types are of decisive importance. To date, the predictors for resilient weed seed and pest regulation by carabids over space and time still have not been clearly identified. Detailed knowledge about the extent to which the availability of different food types can affect food choice is also lacking, especially for omnivorous carabid species. Preliminary findings suggest that the extent of weed seed or slug control may depend on the species composition of the carabid community (Bohan et al. 2011; Jowett et al. 2020; Scaccini et al. 2020). In addition, it is conceivable that the availability of food, which may also change during the year, plays a critical role in sustaining a carabid population for effective weed and pest control (Carbonne et al. 2020).

Currently, carabids are mostly divided into the different trophic-functional groups of carnivores, omnivores and predominantly granivores (Kulkarni et al. 2015). Based on this, more detailed prey categories should be developed so that, for example, subcategories by prey types such as slugs or aphids can be defined or even specific predator–prey species interactions can be identified in a food web approach, highlighting trophic interactions between each carabid species and prey and weed species consumed. There is often a lack of detailed information on the trophic niches of specific carabid species and the driving forces that may influence their food choice. Recent research has focused mainly on the aspect of animal pest control, i.e. on the identification of carabid species that consume invertebrate pests such as aphids, and the effects of the presence or absence of non-pest prey such as earthworms or springtails (Lang 2003; Roubinet et al. 2017, 2018; Staudacher et al. 2016, 2018; Winder et al. 2005). In contrast, little is known about the role of weed seeds in carabid diets and their species-specific seed preferences. This information, however, is highly relevant for the predictive power of ecosystem service provision for both natural pest and weed control (Carbonne et al. 2020). Previous conclusions on weed seed predation were primarily based on the comparison of carabid presence–absence data and changes in the weed seedbank in general (Bohan et al. 2011; Petit and Bohan 2017). Moreover, spatial and temporal patterns of carabid activity-density in cereal fields have not yet been shown to be satisfactory predictors of seed predation alone (Saska et al. 2008). Preference experiments have shown that carabid body mass and species identity as well as seed size all influence seed selection (Honek et al. 2003, 2007; Pocock et al. 2021). Accordingly, it was found that weed seed traits, such as seed size, can affect feeding behaviour and hence food choice of carabids. Additional tests in a further preference experiment clarified that, besides seed mass, lipid content can also play a major role in the weed seed consumption by carabids (Gaba et al. 2019).

Nevertheless, the factors that cause a carabid species to prefer certain seed species over others in the field are not yet fully understood (Talarico et al. 2016). Seasonality may be one of these factors, as both food availability and the presence of different carabid species change over time. Furthermore, there is a lack of knowledge on whether and to what extent weed seed and pest regulation services may be in competition with each other, e.g. through the food choice of omnivorous carabid species. Similarly, the factors that direct carabid food choices towards animal prey or seeds are still unknown.

Molecular diet analyses have proven to be extremely useful tools in unravelling trophic relationships at the species-specific level (King et al. 2008, 2011; Pompanon et al. 2012; Sint et al. 2011; Staudacher et al. 2016) and, not least, to elucidate the dietary choice of carabids (Frei et al. 2019; King et al. 2010). DNA-based results from feeding experiments confirm earlier observations (Honek et al. 2007; Koprdova et al. 2008; Martinkova et al. 2006; Saska et al. 2008) that the carabid *Pseudoophonus rufipes* (De Geer, 1774) feeds on various seed species (Sint et al. 2018; Wallinger et al. 2015). *Pseudoophonus rufipes* is also known to consume eggs and small juveniles of the slug species *Deroceras reticulatum* (Müller, 1774) (El-Danasoury et al. 2017). Likewise, the experiments of Thomas et al. (2009) show that *Pterostichus melanarius* (Illiger, 1798) readily eats juvenile slugs *D. reticulatum* and *Arion intermedius* (Normand, 1852). Although feeding experiments are very helpful for studying food choices and inferring possible food preferences of carabids, they only marginally reflect the situation under field conditions. This is because it is entirely possible that the environmental conditions in the field may affect the carabids in such a way that their feeding behaviour differs from that in a feeding experiment (Charalabidis et al. 2017). Frei et al. (2019) were able to detect plant DNA as evidence of seed predation by carabids in a real-world scenario in cereal fields, but without further identifying seeds to species level.

In summary, there is limited information on the factors influencing the provision of pest and weed control services of carabids in the field. For this reason, we present in the following a field experiment in which we investigated with DNA-based techniques the dietary choice of carabids with regard to animal and seed predation in an organically managed winter wheat field using three different treatments: plots with (I) the addition of slugs, (II) the addition of a weed seed mixture, (III) the addition of both the weed seed mixture and slugs, and (IV) control plots with no treatment. This experiment aimed to examine whether the food choice of carabid species changes under different food availability during a field season. We hypothesized that an increase in seed availability would lead to a reduction in slug feeding and vice versa. Therefore, sentinel prey and seed cards were used to measure predation pressure and prey-specific PCR and NGS-based DNA metabarcoding approaches for the identification of carabid food choices. The main objectives of this field study were to explore (1) the different diets of naturally occurring carabid species regarding animal prey and seeds as food source, (2) potential changes in their food choice during the season, and (3) their food choice depending on the food availability of seeds and slugs.

## Materials and methods

### Field site and study design

**Fig. 1:**
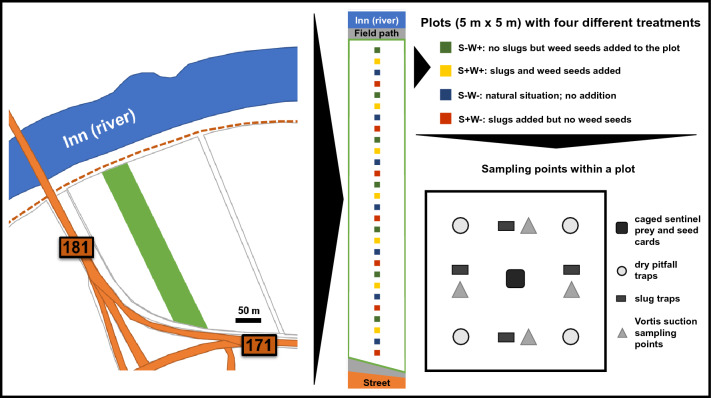
Overview of the field site ‘Rotholz’ (47°23′58.7"N 11°48′26.5"E), located next to the Inn river in Tyrol (Austria); the field strip (green) was divided into 28 plots, each of them receiving one of the four different treatments and all were sampled with four different trap types in the same pattern

The field experiment was established in an organically managed winter wheat field (47°23′58.7"N 11°48′26.5"E) in Rotholz (Tyrol, Austria) between mid-April and mid-July 2017. For this purpose, the field strip was divided into a series of consecutive plots (5 m × 5 m) by fencing each of them with slug barriers (20 cm in height, 5 cm of this within the soil). These slug barriers had the function of preventing the movement of predominantly surface-active invertebrates such as slugs and carabids between plots, but not the movement of arthropods in the vegetation, such as spiders or flying insects that were already in the field. In order to avoid edge effects, the first plot was placed 6 m north of the field edge (a road separated by a hedge), and a distance of 5 m was kept between the individual plots (Fig. 1). Furthermore, the vegetation outside the snail fences was removed to exclude an entry of plant material and creeping arthropods into the experimental area. In doing so, 28 plots were created within the middle of this field strip and distinguished by the following four treatments: (1) slugs+ & weed seeds-, where only slugs were released (TM1: S+W-); (2) slugs- & weed seeds+, where additional weed seeds of a special seed mixture were sown over the plot (TM2: S-W+); (3) slugs+ & weed seeds+, where slugs and weed seeds of the seed mixture were added (TM3: S+W+) and (4) slugs- & weed seeds- as control, where weed seeds from the seedbank and slugs are naturally present (TM4: S-W-). The order of treatments was randomly drawn for the first four plots at the beginning of the field experiment (TM2, TM3, TM4, TM1) and then repeated in the same way seven times in total for all plots (Fig. 1). The seed mixture consisted of a uniform composition of 23 weed species (Table S1). By sowing 15 g of this seed mixture evenly by hand over the designated plots, a seed density of 0.6 g per m^2^ could be achieved in addition to the existing seedbank. In plots without seed addition, field weeds were weeded regularly during the season in order to counteract natural weed growth. Plots with slug addition were supplemented with individuals of grey field slug (*Deroceras* cf. *reticulatum* (Müller, 1774)) and roundback slugs (*Arion* sp. (Férussac, 1819)) to achieve a density of at least one slug per m^2^.

In order to analyse the presence of available prey and to catch the carabids for food analysis, four different types of traps and sampling were prepared in each plot: (1) one cage with a prey and weed card in the centre, (2) four square-placed dry pitfall traps, (3) a Vortis suction sampling took place at four sampling points (196 cm^2^ for each point, 0.0784 m^2^ per plot), and (4) a slug trap next to each of the four Vortis sampling points (Fig. 1). The respective prey and weed cards per cage were prepared for each sampling session as follows: a plastic plant label (10 cm in length, 2 cm in width) was evenly pasted with ten randomly taken seeds of the same seed mixture for preparation of the treatments; the sentinel prey card was pasted with ten meat pieces of a slug of the species *D. reticulatum* or *Arion* sp. instead of seeds. To estimate slug density in the plots, four wooden boards (20 cm long, 15 cm wide, 1.5 cm high) per plot were laid out as slug traps (Archard et al. 2004). Sampling sessions took place on four dates during the season from May to July in 2017 (S1: 2nd May, S2: 2nd June, S3: 27th June and S4: 18th July). For molecular gut content analysis of the carabids, they were collected using dry pitfall traps, each one consisting of a plastic funnel (Ø 7.5 cm, 11 cm in depth) with inserted plastic beakers and covered by a metal roof. To reduce intraguild predation, the plastic beakers were filled up to a maximum of half with wood chips. Each sampling session lasted a total of five days. On the first day of each sampling session, the plots were prepared and closed according to their treatment, with the aim that the carabids in them should move and feed in place, and the cages with their prey and weed cards as well as wooden boards were set up; on the fourth day, the dry pitfall traps were activated and left open for 24 h; and on the fifth day, adult carabids were collected from the dry pitfall traps and put individually in reaction tubes, the remaining cages and traps removed, counting how many slugs were under the wooden boards, and Vortis suction sampling was carried out. Dead carabids were immediately frozen at −24 °C in order to generate whole body extracts, whereas live individuals were stimulated to regurgitate (see Wallinger et al. 2015), sex and species identified (Mueller-Motzfeld 2004) and then released back into the field. DNA extracts from regurgitates were preferred to those from whole body for molecular diet analysis, provided that sufficient numbers were collected.

### Molecular gut content analysis and next-generation sequencing (NGS) run

The DNA extraction and first PCR screening of 1,120 collected individual dietary samples were performed according to the procedure described by Frei et al. (2019). Using this diagnostic multiplex DNA approach, the DNA extracts of the carabids were tested for the food intake of the following prey animals: the three cereal aphid species *Metopolophium dirhodum* (Walker, 1849), *Rhopalosiphum padi* (Linnaeus, 1758) and *Sitobion avenae* (Fabricius, 1775) as pest prey together with springtails (collembolans) and earthworms (lumbricids) as non-pest prey. Targeting the chloroplast *trn*L intron with the primer pair c-B49317 and h-B49466 (Taberlet et al. 1991, 2007) made it possible to screen for plant DNA at the same time. In an additional second screening run, further PCR tests for the slugs *Arion distinctus* (Mabille, 1868), *Arion lusitanicus* (Mabille, 1868), *Deroceras reticulatum* (Müller, 1774) as well as the two families Arionidae and Limacoidea were carried out by analysing the same dietary samples according to the multiplex PCR assay of Guenay-Greunke et al. (2022). Since the *trn*L intron proves to be unsuitable for a species-specific identification of plants or their consumed food material, such as seeds, the second internal transcribed spacer of nuclear ribosomal DNA (*ITS2*) gene region was selected to generate the NGS amplicons. The forward primer UniPlantF2 (5'-GGCACGYCTGYBTGG-3') (Guenay-Greunke et al. 2021) and reverse primer UniplantR (5'-CCCGHYTGAYYTGRGGTCDC-3') (Moorhouse-Gann et al. 2018) were then adapted to set up a sequencing run on an Illumina HiSeq 2500 system (Illumina, San Diego, USA) based on a Nextera DNA library preparation (Guenay-Greunke et al. 2021) and a nested metabarcoding approach (Kitson et al. 2016, 2018). *ITS2* amplicons could be amplified in 601 of the 1120 samples, and only these were sent for sequencing. The NGS run was conducted at the Vienna BioCenter Core Facilities (VBCF) (Vienna, Austria) using a paired-end rapid run mode (2 × 250 bp) with a two-lane rapid flow cell (HiSeq Rapid SBS Kit v2 (500 cycles)). An exact description of the library preparation and the associated NGS run can be found under Guenay-Greunke et al. (2021).

### NGS data processing and filtering

The processing of the paired-end sequencing raw data files obtained from the NGS run was also executed according to Guenay-Greunke et al. (2021). Due to the nested metabarcoding approach applied (Guenay-Greunke et al. 2021; Kitson et al. 2016, 2018), the files of the raw data initially corresponded to a plate sample. The following is an essential overview of the handling of each plate sample file and the bioinformatics workflow with eight steps: the general data quality was controlled by the software FastQC v0.11.8 (Andrews 2010) (step 1); subsequently, the data could be merged directly with the software PEAR v0.9.10 (Zhang et al. 2014) under default settings (step 2); in order to demultiplex the files of the plate samples and thus obtain the individual ones, a specifically written bash script was applied, which worked with the different index combinations including the sequencing errors possible in them and with which only one sample could not be assigned any sequences (step 3); the data trimming was done by removing the adapters and primer sequences using cutadapt v1.18 (Martin 2011), under the settings of a linked adapter option, an error rate of 0.3 and a minimal length requirement of 50 (step 4); for a faster BLAST search, replicates of identical sequences were removed and the copies of them counted by running the application FASTQ/A Collapser of the FASTX-Toolkit v0.0.14 (Gordon and Hannon 2010) (step 5); on the basis of an index hopping threshold of 280 copies per sequence, possible misassigned sequences of a sample were removed by executing a queued awk and echo command at the Linux terminal (step 6); after downloading the nt database (ftp://ftp.ncbi.nlm.nih.gov/blast/db/, status: 25.7.2017), all BLAST+ searches (Camacho et al. 2009) were performed locally by running the BLAST+ software packages v2.8.1 on the high performance compute cluster LEO4 of the University of Innsbruck (step 7), so that finally the species were identified and listed (step 8). The *ITS2* gene region offers the possibility to identify plant species in high resolution and only with one instead of two primer pairs, but it also amplifies fungal and bacterial DNA sections. Such sequences occurred in some gut content samples and were then filtered out. In individual cases, there was evidence of woody plants planted in the surrounding area and of winter cereals, which was also excluded for the analysis of consumed weed seeds within the plots.

### Data evaluation and statistical analyses

To obtain the ratio of animal to plant food for each of the eight carabid species (Table 1), the molecular presence–absence data of the 1,120 gut content samples were assigned to the respective carabid species and the corresponding prey. Based on the number of PCR and NGS detections for each food type and the total numbers of each carabid species, the DNA detection rates were calculated. The resulting food web was then plotted using the graphic software tool “Food Web Designer 3.0” (Sint and Traugott 2016) (Fig. S1). All further statistical analyses and visualisations of the associated data were conducted in R version 4.1.2 (R Core Team 2021) using the packages “xlsx” (Dragulescu and Arendt 2020), “vegan” (Oksanen et al. 2020), “tidyverse” (Wickham 2021), “ggplot2” (Wickham et al. 2021), “patchwork” (Pedersen 2020), “cowplot” (Wilke 2020), “ggpubr” (Kassambara 2020) and “selectapref” (Richardson 2020).

**Table 1:** Number of samples of the eight analysed carabid species

Scientific name	Number of samples
*Agonum muelleri* (Herbst, 1784)	184
*Bembidion tetracolum* (Say, 1823)	184
*Carabus granulatus* (Linnaeus, 1758)	184
*Clivina fossor* (Linnaeus, 1758)	60
*Poecilus cupreus* (Linnaeus, 1758)	184
*Pseudoophonus rufipes* (De Geer, 1774)	47
*Pterostichus anthracinus* (Panzer, 1795)	93
*Pterostichus melanarius* (Illiger, 1798)	184

**Fig. 2:**
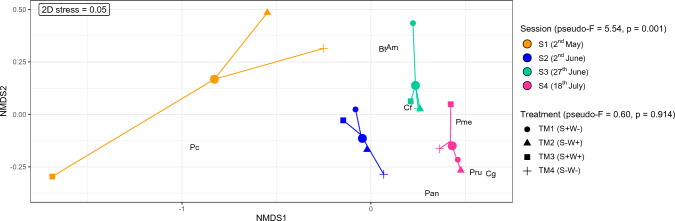
Non-metric multidimensional scaling (NMDS) of carabid community composition showing seasonal influences in terms of the attractiveness of the whole site, not individual plots, on the eight individual carabid species (*Agonum muelleri* (Am), *Bembidion tetracolum* (Bt), *Carabus granulatus* (Cg), *Clivina fossor* (Cf), *Poecilus cupreus* (Pc), *Pseudoophonus rufipes* (Pru), *Pterostichus anthracinus* (Pan), *Pterostichus melanarius* (Pme))

**Fig. 3:**
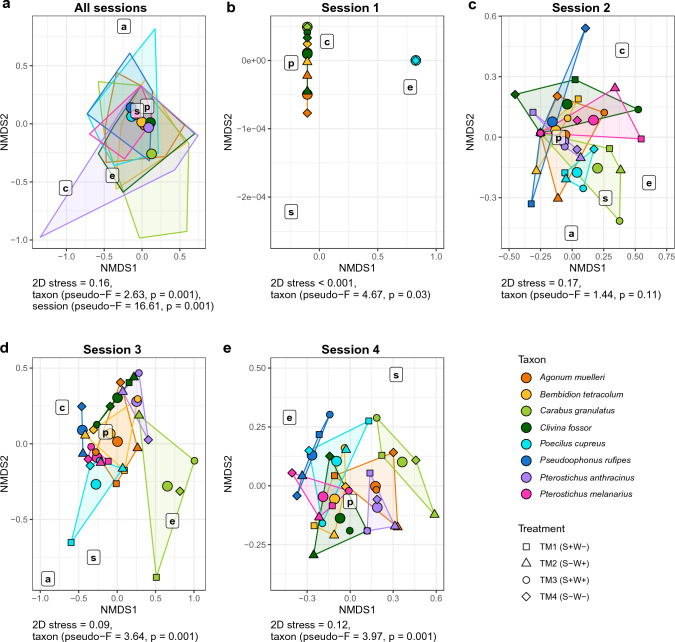
Non-metric multidimensional scaling (NMDS) ordination of prey composition for the eight carabid species based on Bray–Curtis dissimilarity (prey: aphids (a), collembolans (c), earthworms (e), plants (p), and slugs (s)). Each polygon with its corresponding colour represents one of the carabid species. The respective centroids are illustrated by the large dots and the smaller border dots stand for the individual treatments. The ordination data of all sessions **a** were then split based on the four different sampling sessions (**b** session 1 on 2nd May, **c** session 2 on 2nd June, **d** session 3 on 27th June and **e** session 4 on 18th July) and these also show the prey consumption of all carabid species for the corresponding treatment (TM) (TM1: S+W-, TM2: S-W+, TM3: S+W+ and TM4: S-W-)

**Fig. 4:**
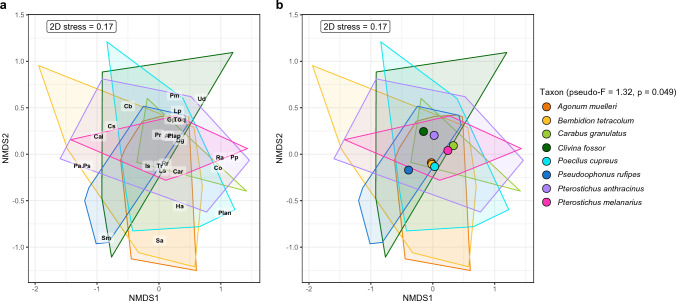
Non-metric multidimensional scaling (NMDS) ordination of the 25 most common plant species whose DNA was found in regurgitates of carabid beetles, based on Bray–Curtis dissimilarity. Each polygon with its corresponding colour represents one of the carabid species. The same data are presented in the form of polygons, with panel **a** representing the 25 plant species and panel **b** the centroids of the carabid species with the large dots. A detailed naming of the individual plant species can be found in the list of abbreviations

**Fig. 5:**
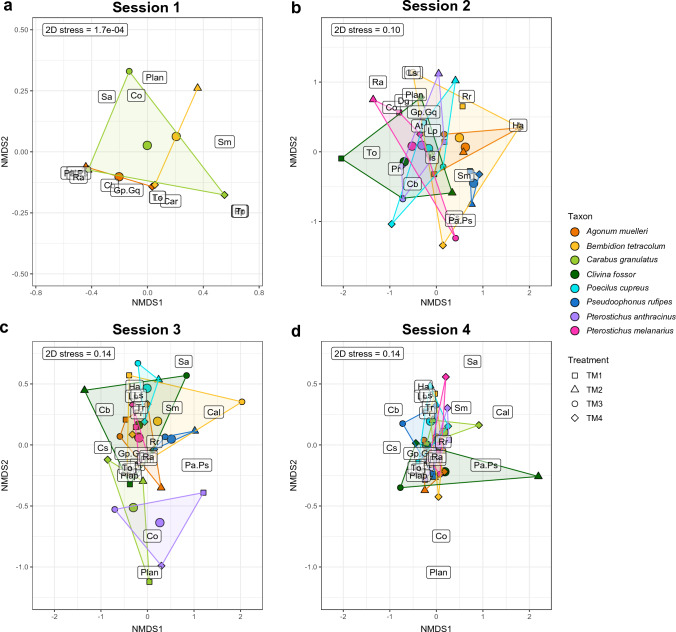
Separation of the NMDS ordination of Fig. 4 based on the four different sampling sessions (**a** session 1 on 2nd May, **b** session 2 on 2nd June, **c** session 3 on 27th June and **d** session 4 on 18th July) showing prey consumption of all carabid species for the corresponding treatment (TM) (TM1: S+W-, TM2: S-W+, TM3: S+W+ and TM4: S-W-). A detailed naming of the individual plant species can be found in the list of abbreviations

**Fig. 6:**
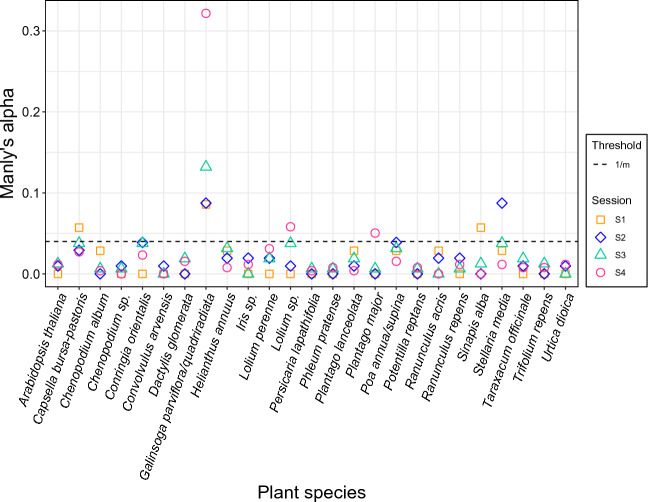
Manly's alpha preference indices for the most detected plant species (*m* = 25) after evaluation of the plant sequences in the gut contents using the next generation sequencing method. Exceeding individual points above the 1/*m* threshold (dashed line at 0.04) indicates preferential consumption of the food for the respective sampling session (S1: 2nd May, S2: 2nd June, S3: 27th June and S4: 18th July)

A first model, performing a permutational multivariate analysis of variance (PERMANOVA), tested whether seasonal effects or the attractiveness of the plots due to the characteristics in the different treatments could explain differences in food choice related to carabid community composition (Fig. 2). The objective here was to test whether seasonality and the different treatments induce formative differences in community composition and thus explain differences in food choice. Further equal models (PERMANOVA) were used again to calculate whether the eight carabid species, the different sampling sessions (S1-S4) and treatments (TM1-TM4) would explain possible differences in prey type detection rates (split per session and treatment) after PCR analysis of the gut content samples. Due to the low detection rates for aphid and slug species, the rates of the individual species were combined as aphids and slugs, respectively. The results of these PERMANOVA tests were then illustrated with a non-metric multidimensional scaling (NMDS) ordination of prey composition (R packages “vegan” and “tidyverse” under the conditions: adonis function under Bray–Curtis dissimilarity calculation and 999 permutations as well as metaMDS under *k* = 2 and 999 permutations). Since the differences in prey detection rates could be explained by seasonal effects, the total data were divided among the four different sampling sessions and each of these was statistically analysed identically to the total data (Fig. 3). In addition, the three factors carabid taxon, sampling session and treatment were explicitly tested for differences in the detection rates of the most frequently identified plant species (*n* = 25), again calculated with PERMANOVA and NMDS. This was done both for the whole dataset (Fig. 4) and for each individual session (Fig. 5). After identifying the plant species in the NGS data, only those ones that could be detected in at least five gut content samples of the carabids served as the data basis for this, which ended up in 25 plant species (see Table S3). For these selected plant species, the individual Manly's alpha preference indices (Manly 1974) (R package “selectapref”) were then calculated per sampling session (Fig. 6). Index values above the set 1/*m* threshold (here: 0.04 as *m* = 25, the number of more common plant species) can be ascribed to higher detection rates in the gut contents. Consequently, they indicate a possible choice preference of carabids for specific seed species.

The percentages of seeds or slug tissue pieces removed from the sentinel prey cards served as a quantitative measure of seed and slug predation in each plot. Using Pearson correlations, it was tested whether there was a linear relationship between the removal of slug pieces or seeds from the sentinel prey cards and the detection rates of the different food types in the corresponding plots (Fig. S2). Likewise, the correlation between the count data of the collembolans from the Vortis samples and the detection rates of their DNA from the molecular gut content analysis was calculated (Fig. S3). To assess the availability of naturally occurring seeds in the different plots, they were extracted from the Vortis samples and identified to species level using the Seed Information Database (SID) (Royal Botanic Gardens Kew 2008) (Table S1). This allowed a comparison of the seed species derived from the Vortis samples with the sown seeds of the seed mixture for treatments 2 and 3 and at the same time a check if these Vortis seed species could also be found within the NGS samples (Table S1).

## Results

### The food choice of carabids

Field sampling allowed a molecular gut content analysis of 1,120 samples from eight different carabid species (Table 1). In the diagnostic PCR screening for the differentiation of animal food and consumed seeds, 72% of all detections were plant DNA, followed by 12.5% lumbricid DNA, 8.3% collembolan DNA and 2.0% for the cereal aphids (0.9% *R.* *padi*, 0.6% *M. dirhodum*, and 0.5% *S.* *avenae*) as well as 4.1% for slugs (1.6% Limacoidea, 1.4% Arionidae, 1.1% *A.* *lusitanicus*, and 0.8% *D.* *reticulatum*). The slug species *A.* *distinctus* was absent in the gut content samples (Fig. S1). Seven of the eight carabid species examined consumed slugs, which are *Agonum muelleri* (Herbst, 1784), *Bembidion tetracolum* (Say, 1823), *Carabus granulatus* (Linnaeus, 1758), *Poecilus cupreus* (Linnaeus, 1758), *Pseudoophonus* *rufipes* (De Geer, 1774), *Pterostichus anthracinus* (Panzer, 1795), *Pterostichus* *melanarius* (Illiger, 1798), and only *Clivina* *fossor* (Linnaeus, 1758) did not (Table 1 and Fig. S1). The food choices of the different carabid species in terms of the ratio between animal food and consumed seeds were similar for all of them (Fig. S1): plant detection rates were much higher than those of animal prey, ranging between 65 and 84%, with the exception of *C. granulatus* having a clearly lower percentage (47%). Compared to these high plant detection rates, the next highest value for animal prey was only at 17% for lumbricids when looking at the beetle species *C. granulatus* or *P. cupreus* (Fig. S1).

Calculating the Pearson correlation between seed removal rates from seed cards and plant DNA detection rates, a positive linear relationship was observed (*t* = 2.752, *df* = 97, *r* = 0.269, *p* = 0.007) (Fig. S2). Similarly, the abundance of collembolans was positively correlated with the detection rates of their DNA in the gut content analysis of carabids (Pearson correlation,* t* = 6.426, *df* = 97, *r* = 0.546, *p* < 0.001) (Fig. S3). However, there was no correlation between the removal rates of slug pieces on the sentinel prey cards and the detection rates of slug DNA (*p* = 0.747) (Fig. S2). In fact, most of the slug pieces on the sentinel prey cards were not consumed at all (Fig. S2). Moreover, no slugs were below the wooden boards that were intended as slug traps, indicating a very low density.

The NGS results of the 601 samples sent for sequencing after positive plant screening showed a broad spectrum of consumed weed seeds, comprising 80 plant species from 17 different orders (Table S2 and S3). Most of these species (55 species; 69%) were present in only a few gut content samples (≤ 4), suggesting a random consumption. For the 25 most frequently detected seed species, prey preference was investigated via Manly's alpha preference indices. Based on this, preferred consumption was found for the following eight plant species: *Capsella bursa-pastoris* ((L.) Medik., 1792), *Conringia orientalis* ((L.) Dumort.), *Galinsoga parviflora/quadriradiata* (Cav., Ruiz & Pav.), *Lolium* sp., *Plantago major* (L.), *Poa annua/supina* (L., Schrad.), *Sinapis alba* (L.) and *Stellaria media* ((L.) Vill.) (Fig. 6). Of these, *G. parviflora/quadriradiata* was the most frequently detected plant species of all gut content samples and more often present per carabid species as well as sampling session compared to all the other seed species (Table S2 and S3). The consumption of the other seven plant species listed above was restricted to a specific sampling time point, such as *C. bursa-pastoris* and *S. alba* in May (S1), *C. orientalis*, *P. annua/supina* and *S. media* in June (S2 and/or S3), and *Lolium* sp. and *P. major* in July (S4). In the Vortis samples, twelve plant species could be identified, with only two of them being absent in the NGS data: *Elymus repens* ((L.) Gould) and *Papaver rhoeas* (L.) (Table S1).

### The effect of species identity and seasonality on the food choice of carabid species

Carabid community composition changed significantly over the entire sampling period (season: *R*^2^ = 0.60, *F* = 5.54, *p* = 0.001). The observed changes can be mainly attributed to species such as *C. granulatus*, *P. rufipes*, *P. anthracinus* and *P. melanarius*, which were caught more frequently in July (S4) than in the earlier sessions (Fig. 2). In contrast, *P. cupreus* was quite abundant and evenly collected across all four sampling periods.

When comparing animal and seed diets based on diagnostic PCR in an NMDS ordination, food choice was significantly different for each carabid species and during the season for all prey types (taxon: *R*^2^ = 0.16, *F* = 2.63, *p* = 0.001, season (sampling sessions): *R*^2^ = 0.33, *F* = 16.61, *p* = 0.001) (Fig. 3). The same was true for the 25 most frequently detected seed species of the NGS data analysis (taxon: *R*^2^ = 0.10, *F* = 1.32, *p* = 0.049, season: *R*^2^ = 0.15, *F* = 5.23, *p* = 0.001) (Fig. 4).

Looking at the NMDS ordination of all sampling sessions (Fig. 3a), *C. granulatus* clearly separated itself as a species from the other carabids, as this beetle species consumed proportionally more animal prey than plant food relative to the other carabid species. Compared to the individual sessions (session 1 to 4), this was particularly obvious in the evaluation of all sessions (Fig. 3). However, this difference disappeared when only the consumption of the 25 most common seed species was considered: here, *C. granulatus* resembled the other carabid species in its food choice (Figs. 4, 5). With regard to the 25 most frequently detected seed species, the food choice of all carabid species was similar, with the exception of *P. rufipes* (Fig. 4). The gut content analysis of *P. cupreus* resulted in a detection rate of 64% for DNA from 24 of these 25 plant species (see Table S2), indicating that comparatively few other seed species had been consumed. For *C. granulatus* as another example, it was 47% and 17 of the 25 species. DNA of *G. parviflora/quadriradiata*, the most frequently detected and a non-provided species, was identified with a maximum frequency in *C. granulatus* (35.5%) and a minimum in *P. cupreus* (21%).

The pattern of food choice also varied greatly within carabid species during the season (Figs. 3,  5). In the case of *B. tetracolum*, for example, the detection rate for collembolans was 65% in May and decreased to 3% towards July and increased from 56 to 80% for plants. In general, the detection rates of collembolans showed a decrease from May (S1) to July (S4) (see Fig. S3). In contrast, the detection rates for *G. parviflora/quadriradiata* gradually increased towards the last sampling session in July (S4) (Fig. 6 and Table S3).

### Effects of treatments on the food choice of carabids

The individual treatments had no detectable effect on the carabid community composition (treatment: *R*^2^ = 0.14, *F* = 0.60, *p* = 0.914) (Fig. 2). Likewise, the NMDS ordinations based on their diet (diagnostic PCR and NGS) did not show any statistically significant differences in food choice attributable to the four treatments (PERMANOVA for the prey composition data:* p* = 0.87 (Fig. 3) and for those of the 25 plant species: *p* = 0.988 (Fig. 4)). Fourteen of the 80 plant species identified by NGS were species that also corresponded to the seed mixture (Table S3), with four of these being among the 25 most frequently detected plant species in the carabid diet (Fig. 6 and Table S3): *S. media*, *C. bursa-pastoris*, *P. annua/supina*, *Taraxacum officinale* (F. H. Wigg.). Three of them were present in the seed mixture as well as in the gut content samples, but also in Vortis samples from plots without added seeds (TM1 and TM4) (Table S1), i.e., *S. media*, *C. bursa-pastoris* and *Poa* sp. This indicates that these weeds were already widespread everywhere in the field. *Galinsoga* *parviflora/quadriradiata*, the most frequently detected species in the gut content samples, was not included in the seed mixture.

## Discussion

The main objective of this study was to obtain detailed information on the food choice of different carabid species across the season and under different availability of slugs and weed seeds. New insights would be extremely helpful in understanding which carabid species provide regulatory services, and when and if these services may be in competition. The results indicate a strong impact of both the carabid species identity and the season, while carabid food choice seems to be independent of the addition of weed seeds and slugs. This suggests at present that food choice was independent of the availability of the different food items and that the control of weed seeds and slugs did not compete with each other.

Earlier studies have shown that *P. cupreus* (Oberholzer et al. 2003; Oberholzer and Frank 2003), *P. rufipes* (Ayre 2001) and *P. melanarius* (Ayre 2001; Bohan et al. 2000; McKemey et al. 2003; Oberholzer et al. 2003; Oberholzer and Frank 2003) can rightly be considered as natural predators of slugs. Also in this experiment, all carabid species except *C. fossor* had consumed slugs. However, with a total of 4.1% of slug detections, the consumption of this prey type was generally low, regardless of the treatment. The low predation rates in this study were likely due to the low slug densities. Furthermore, the density of at least one slug per m^2^ in the treated plots might have been too low to show any effect. Therefore, we recommend to increase the number of slugs and also to add other pest species in future experiments in order to investigate the competition between weed seed and pest control in more detail. Moreover, our findings indicate that there may be less linear relationships between food availability and dietary choice than indirect effects. Obviously, the increased availability of a specific food type does not necessarily entail more seeds being eaten. However, in our analysis we found that the change of the availability of food sources seemed to have more of a network effect, leading to shifts in the entire food web. In our experiment, hardly any slug pieces were consumed from sentinel prey cards, indicating that the carabids may have preferred food sources other than slugs. It should be noted that the knowledge gained from the sentinel prey cards can only give an indication of the feeding preferences of consumers, as the results of work with sentinel prey or the predation rates determined from them may be the same as those from predation on real prey but may also be different (Gossner et al. 2020). In this sense, the sentinel prey cards cannot reflect the full range of real prey available in the field. In contrast to the slugs, the generally high detection rates of plant DNA suggest that seed predation might have played a major role in the diet of the carabid species in cereal fields.

In molecular diet studies, it is generally important to verify that the detected DNA comes directly from the prey rather than from any other potential source. This is especially the case for plant DNA, which can influence the analysis of gut content samples in the form of environmental DNA (eDNA). Unfortunately, it is usually not possible to completely exclude the possibility that traces of eDNA may also have entered a sample. For example, pollen and minute amounts of soil material can stick to the body surface of carabids. In this context, it should be taken into account that sufficient material and special DNA extraction procedures are required for DNA extraction from pollen, e.g. to enable the pollen grain walls to be broken open (Johnson et al. 2019; Kelley et al. 2020; Kraaijeveld et al. 2015), which makes it unlikely that much pollen DNA was present in regurgitate extracts of this study. Moreover, the positive correlation between seed card removal rates and plant DNA detection rates in this study, as well as the match of the detected plant species with the seeds in the Vortis samples, indicate that these detections can be mainly ascribed to feeding events. The same holds true for a possible influence on the results due to secondary predation, meaning the detection of plant food consumed by herbivores that the carabid has eaten. But as previous feeding experiments have demonstrated, this seems to be a rather negligible factor (Guenay et al. 2020).

The results of the present study were based on an experiment in a single field with only one winter cereal variety in one region. Even if this allows initial conclusions to be drawn about possible temporal changes in weed and pest regulation by carabids in winter wheat, further studies in this direction are needed in order to be able to derive generally valid patterns. To account for spatial differences, more studies of this kind in various geographical regions will be required. This is because their findings should lead to a better understanding of the underlying patterns and factors providing the resilience of these ecosystem services, which are crucial for sustainable agriculture in the future. In this context, it would be essential to also consider the aspect of landscape complexity, as it has been shown that the heterogeneity of landscape structures on agricultural land has a strong influence on what happens in the fields (e.g. rich versus poor of semi-natural habitats, etc.) (Fischer et al. 2011; Rusch et al. 2013; Holland et al. 2016). With the DNA-based diet analysis techniques used here, numerous gut content samples could be analysed. This semi-quantitative approach, generating presence–absence data, provided information on which carabid species from the winter wheat field consumed the prey and seeds tested. In addition, a strong impact of carabid species identity on food choice rather than the varying availability of slugs and weed seeds became apparent. Since carabid identity seems to play a crucial role in slug and seed predation, it would be interesting in the future to investigate in more detail which of the carabid species consume particularly large numbers of pests or weed seeds. Therefore, a quantitative approach to assess the number of each species eaten would be helpful to identify the carabid species that are most effective in biocontrol of a particular pest or weed species.

Among the samples that tested positive for plant DNA, a wide variety of plant species was identified by NGS. Here, the *ITS2* gene proved to be a well-suited target region for metabarcoding, which largely enabled the identification of weed species down to the species level. In doing so, the seed species mainly reflected those which were present in the Vortis samples and therefore also in the field. Since there was no treatment effect from the extra seeds sown, this additional food source seemed to have had little impact on the feeding habits of the carabids. The high number of matches between the molecularly identified plant species within the food samples and the naturally occurring seed species represented by the Vortis samples further illustrates that the carabids predominantly consumed seeds that were already present in the field. This indicates that the naturally occurring food supply from seeds on site was sufficient and diverse enough for each carabid species, so that the addition of seeds did not alter their dietary choice. The generally high proportion of plant DNA in carabid guts was thereby consistent with former findings demonstrating the importance of weed seeds as a food source (Frei et al. 2019). Earlier indications of a possible regulatory effect of carabids on the weed seedbank (Bohan et al. 2011; Carbonne et al. 2020) were thus confirmed as well.

Some of the seed species identified as carabid food are already known to cause serious problems as weeds in agriculture. Of the 25 most frequently detected species, the grass *Lolium* sp. is often sown as an intermediate culture and can establish itself as a field weed in subsequent crops (Bayerische Landesanstalt für Landwirtschaft (LfL) 2022). *Galinsoga* sp. provides considerable competition for nutrients and water due to the dense and deep root system (up to 80 cm), which also applies to *P. major* due to its rapid spread and strong roots. *Sinapis* sp. is known as a host plant for fungal pathogens and insect pests (Badenes-Perez 2019; Bugg et al. 2008; Rouxel and Balesdent 2005). *Stellaria media* is another nitrophilic weed which competes strongly for nutrients, space, water, and light, particularly in mass growth, and can promote the microclimate for fungal infections (Gehring and Thyssen 2019). With *P. annua*, a widespread, small-growing grass with low competitive strength (Gehring and Thyssen 2019), and the herbaceous *C. bursa-pastoris*, economically less relevant species were also among them.

The results of our study show that the species identity of carabids is of great importance for a more detailed understanding of the observed feeding interactions. Previous studies classified the carabid species *B. tetracolum*, *C. granulatus*, *C. fossor* and *P. anthracinus* as carnivorous (Brooks et al. 2012; Cardarelli and Bogliani 2014; Kirichenko-Babko et al. 2020) and *P. cupreus* as omnivorous (Brooks et al. 2012; Cardarelli and Bogliani 2014). In the case of *A. muelleri* and *P. melanarius*, no clear classifications existed until now, as both species were described as either carnivorous (Cardarelli and Bogliani 2014) or omnivorous (Brooks et al. 2012; Kirichenko-Babko et al. 2020; Lucas and Maisonhaute 2015), depending on the literature referred to. The same is true for *P. rufipes*, which was sometimes classified as a granivore (Kirichenko-Babko et al. 2020) or even an omnivore (Brooks et al. 2012; Cardarelli and Bogliani 2014). Based on the NMDS analysis, *C. granulatus* was the only carabid species that strongly differed from the other carabids and was identified as a carnivore. The other carabid species which were originally classified as carnivorous (*B. tetracolum*, *C. fossor*, and *P. anthracinus*) had a surprisingly high proportion of plant DNA detections, allowing them to be classified as omnivores. The high detection frequencies for plant DNA in *A. muelleri* and *P. melanarius* also support the theory that they consumed seeds and are consequently omnivorous. Similarly, the dietary habits of *P. cupreus* corresponded to the ones of an omnivorous consumer. *Pseudoophonus rufipes*, on the other hand, was characterized as an omnivore with a stronger tendency towards granivory. Since the carabids are a large family with a global geographic distribution and the diet spectrum within this family can be very diverse, it is consequently important to check exactly for the respective species in which feeding guild it should be classified.

In addition to the carabid species identity, the different sampling sessions were found to have a significant effect on dietary choice, indicating that seasonality plays an important role. As exemplified by *B. tetracolum*, the plant detection rate in carabid guts generally increased towards summer, when flowering and seed shedding predominate. This is in accordance with Honek et al. (2006), who described increased seed consumption by carabids from spring to summer. Seasonal differences were again observed in the composition of the carabid community itself. This reflects the different life cycles of the individual carabid species, being either spring or autumn breeders (Kotze et al. 2011; Lovei and Sunderland 1996; Luff and Larsson 1993). Species emerging earlier in the year often have broader trophic niches because they lack rich feeding opportunities at that time. Characteristic of this is the food choice of *P. cupreus*, which is usually one of the earliest carabids appearing in the season and also here showed a high activity density in May (S1) as well as a high flexibility in food choice. Other carabid species, in contrast, were mainly caught in summer (S3 and S4). Reasons for the growing carabid presence could be the food availability in the form of newly developed seeds and an increasing abundance of animal prey including pests (e.g. aphids).

The observed feeding preference of carabids for seeds of the species *C. bursa-pastoris*, *G. parviflora*, *P. major*, *P. annua* and *S. media* was in accordance with previous findings on their food choice (Honek et al. 2003, 2007; Saska et al. 2008). *Capsella* *bursa-pastoris*, *G. parviflora/quadriradiata* and *S. alba*, for example, have a longer flowering period during the season, which may explain the higher Manly's alpha values. Additionally, it was noted that the feeding preference for seeds of summer-flowering plants such as *C. orientalis* and *P. annua/supina* and late-flowering plants such as *P. major* could be seen right in the corresponding period of ready seed formation.

In conclusion, our study shows that both the carabid species identity and seasonality have a strong impact on their food choice and hence on the provision of their ecosystem services such as pest (e.g. aphids and slugs) and weed seedbank regulation in cereal fields. The importance of carabid species identity in driving dietary choices might be useful to promote suitable carabid species against specific pest or weed problems. In this field experiment, a broad variety of weed seeds was found to be consumed, with some of them being detected more frequently than others. The availability of weed seeds and slugs did not seem to have any effect on the food choice of carabids. Therefore, it was not possible to draw conclusions on whether the ecosystem services of weed and slug control were in competition with each other or not. This may require more detailed research in the future, such as large-scale studies at different landscape levels where carabids are collected from several fields in various regions. The nature and complexity of the landscape has a strong effect on the diversity and abundances of predators and pests, as well as on seed availability and thus on what happens in the fields. Accordingly, it is not enough to consider what is going on in the fields, but to look at the whole beyond the field boundaries at the landscape level and also to compare it. This would be helpful for a better understanding of the ecosystem services carabids provide. For such large-scale studies, the molecular methods presented here are ideally suited, as large numbers of individual samples can be analysed quickly without having to kill these beneficial organisms. If field experiments were planned for several years, it would also be possible to measure whether and to what extent the damage caused by weeds and pests in the field is reduced by the feeding behaviour of the carabids over the years and how this affects yield. However, the results clearly demonstrate that carabids appear to occupy broader trophic niches than previously thought, and that seeds are a welcome food source for most of the species studied here, thus highlighting their potential for both weed seed and pest control in arable land.

## Author contributions

DB, MT and CW conceived and designed the study. YGG, HT and CW performed the field sampling. HT was responsible for DNA extractions and PCR screening. YGG performed the NGS library preparation and bioinformatic analysis of NGS data, including its evaluation. YGG and CW wrote the manuscript, which all authors revised and approved for submission.

### Supplementary Information

Below is the link to the electronic supplementary material.

Supplementary file1 (DOCX 477 kb)

## Data Availability

Further information, especially a more detailed list of the 80 plant species amplified in the NGS samples, can be found in the supplements of this article. All the relevant NGS data (analysed reads) are available in the ‘Open Science Framework (OSF)’ storage of the project ‘Trophic assessment of ecosystem services provided by carabid beetles in agricultural land’ in the folder ‘Rotholz_data’, accessible under the 10.17605/OSF.IO/YQSTJ via https://osf.io/yqstj/.

## Plants

At: *Arabidopsis thaliana*
Cal: *Chenopodium album*
Car: *Convolvulus arvensis*
Cb: *Capsella bursa-pastoris*
Co: *Conringia orientalis*
Cs: *Chenopodium* sp.
Dg: *Dactylis glomerata*
Gp/Gq: *Galinsoga parviflora/quadriradiata*
Ha: *Helian* *thus* *annuus*
Is: *Iris* sp.
Lp: *Lolium perenne*
Ls: *Lolium* sp.
Pa/Ps: *Poa annua/supina*
Plan: *Plantago lanceolata*
Plap: *Persicaria lapathifolia*
Pm: *Plantago major*
Pp: *Phleum pratense*
Pr: *Potentilla reptans*
Ra: *Ranunculus acris*
Rr: *Ranunculus repens*
Sa: *Sinapis alba*
Sm: *Stellaria media*
To: *Taraxacum officinale*
Tr: *Trifolium repens*
Ud: *Urtica dioica*

## Carabids

Am: *Agonum muelleri*
Bt: *Bembidion tetracolum*
Cf: *Clivina fossor*
Cg: *Carabus granulatus*
Pan: *Pterostichus anthracinus*
Pc: *Poecilus cupreus*
Pme: *Pterostichus melanarius*
Pru: *Pseudoophonus rufipes*
