# Supplementary material for: Consumer identity but not food availability affects carabid diet in cereal crops
Source: J Pest Sci (2004). 2023 Apr 24;97(1):281–96. doi: 10.1007/s10340-023-01620-w (PMC10784395; doi:10.1007/s10340-023-01620-w)
Supplement: Supplementary file 1 — Supplementary file1 (DOCX 477 kb) [file 10340_2023_1620_MOESM1_ESM.docx]

**Supporting information**

Additional Supporting Information may be found in the online version of this article.

**Supplementary tables**

Table S1: List of the seed mixture consisting of the 23 field seeds, which are typically found on agricultural land in this region and were added for treatment 2 (S-W+) and 3 (S+W+) (orange bars), and of seeds detected within the Vortis samples (blue bars); the bars indicate the presence of the respective plant species; in addition, it is indicated if one of the plant species of the weeds appeared in the reads of carabid gut content samples (green bars).

| **Plant species in** | **seed mixture** | **Vortis samples** | **NGS reads of DNA samples** |
| --- | --- | --- | --- |
| *Alopecurus myosuroides* (Huds.) |  |  |  |
| *Apera spica-venti* ((L.) P. Beauv., 1812) |  |  |  |
| *Avena fatua* (L.) |  |  |  |
| *Bromus hordeaceus* (L.) |  |  |  |
| *Bromus sterilis* (L.) |  |  |  |
| *Capsella bursa-pastoris* ((L.) Medik., 1792) |  |  |  |
| *Cardamine hirsuta* (L., 1753) |  |  |  |
| *Cerastium fontanum* (Baumg.) |  |  |  |
| *Chenopodium album* (L.) |  |  |  |
| *Elymus repens* ((L.) Gould) |  |  |  |
| *Galeopsis tetrahit* (L.) |  |  |  |
| *Galinsoga quadriradiata* (Ruiz & Pav.) |  |  |  |
| *Galium aparine* (L.) |  |  |  |
| *Geranium dissectum* (L.) |  |  |  |
| *Lamium purpureum* (L.) |  |  |  |
| *Lolium multiflorum* (Lam., 1779) |  |  |  |
| *Myosotis arvensis* ((L.) Hill, 1764) |  |  |  |
| *Papaver rhoeas* (L.) |  |  |  |
| *Poa* sp. |  |  |  |
| *Poa* *annua* (L.) |  |  |  |
| *Polygonum aviculare* (L.) |  |  |  |
| *Polygonum convolvulus* (L.) |  |  |  |
| *Sinapis arvensis* (L.) |  |  |  |
| *Stellaria media* ((L.) Vill.) |  |  |  |
| *Taraxacum officinale* (F. H. Wigg.) |  |  |  |
| *Thlaspi arvense* (L.) |  |  |  |
| *Tripleurospermum inodorum* ((L.) Schultz Bip.) |  |  |  |
| *Veronica* sp. (L.) |  |  |  |
| *Vicia hirsuta* ((L.) Gray) |  |  |  |
| *Viola arvensis* (Murray) |  |  |  |

Table S2: List of the 80 plant species that were amplified in the NGS samples; divided according to carabid species (*Agonum muelleri* (*Am*), *Bembidion tetracolum* (*Bt*), *Carabus granulatus* (*Cg*), *Clivina fossor* (*Cf*), *Poecilus cupreus* (*Pc*), *Pseudoophonus rufipes* (*Pru*), *Pterostichus anthracinus* (*Pan*), *Pterostichus melanarius* (*Pme*)); plant species that were included in the seed mixture for treatment 2 and 3 were highlighted in bold.

| **Order** | **Family** | **Plant species** | ***Am*** | ***Bt*** | ***Cg*** | ***Cf*** | ***Pc*** | ***Pru*** | ***Pan*** | ***Pme*** | **Sum** |
| --- | --- | --- | --- | --- | --- | --- | --- | --- | --- | --- | --- |
| Asterales | Asteraceae | *Galinsoga parviflora/quadriradiata* | 38 | 32 | 27 | 10 | 27 | 16 | 18 | 48 | 216 |
|  |  | *Helianthus annuus* | 8 | 9 | 3 |  | 6 |  | 1 | 4 | 31 |
|  |  | ***Taraxacum officinale*** | 2 | 2 | 3 | 2 | 2 |  | 1 | 5 | 17 |
|  |  | *Achillea millefolium* |  | 1 | 1 |  |  |  |  | 1 | 3 |
|  |  | *Leontodon hispidus* |  |  | 2 |  | 1 |  |  |  | 3 |
|  |  | *Matricaria chamomilla* |  | 1 |  |  |  | 1 |  |  | 2 |
|  |  | *Artemisia vulgaris* | 1 |  |  |  |  |  |  |  | 1 |
|  |  | *Bellis perennis* |  |  |  |  |  |  |  | 1 | 1 |
|  |  | *Cirsium* sp. |  |  | 1 |  |  |  |  |  | 1 |
|  |  | *Crepis* sp. |  |  |  |  |  |  |  | 1 | 1 |
|  |  | *Erigeron canadensis* |  |  |  |  |  |  |  | 1 | 1 |
|  |  | ***Tripleurospermum inodorum*** |  |  |  |  |  | 1 |  |  | 1 |
| Caryophyllales | Caryophyllaceae | ***Stellaria media*** | 5 | 2 | 2 | 4 | 13 | 7 | 2 | 5 | 40 |
| Poales | Poaceae | *Lolium* sp. | 4 | 7 | 4 | 1 | 5 | 5 | 3 | 10 | 39 |
|  |  | ***Poa annua/supina*** | 6 | 2 | 1 | 2 | 4 | 5 | 1 | 2 | 23 |
|  |  | *Lolium perenne* | 3 | 3 |  | 2 | 4 | 1 | 1 | 7 | 21 |
|  |  | *Dactylis glomerata* | 1 | 4 | 3 |  | 1 |  | 1 | 3 | 13 |
|  |  | *Phleum pratense* | 1 |  | 1 |  | 2 |  | 2 |  | 6 |
|  |  | *Alopecurus pratensis* |  |  |  |  | 2 | 1 |  | 1 | 4 |
|  |  | *Trisetum flavescens* | 1 | 1 |  |  |  | 2 |  |  | 4 |
|  |  | *Poa trivialis* |  | 1 |  |  | 1 |  |  | 1 | 3 |
|  |  | ***Apera spica-venti*** |  | 1 |  |  |  | 1 |  |  | 2 |
|  |  | *Arrhenatherum elatius* |  |  | 1 |  | 1 |  |  |  | 2 |
|  |  | *Agrostis* sp. |  |  |  |  |  |  |  | 1 | 1 |
|  |  | *Festuca pratensis* |  |  |  | 1 |  |  |  |  | 1 |
|  |  | *Festuca rubra* |  |  | 1 |  |  |  |  |  | 1 |
|  |  | *Poa pratensis* | 1 |  |  |  |  |  |  |  | 1 |
| Brassicales | Brassicaceae | ***Capsella bursa-pastoris*** | 8 | 3 | 3 | 2 | 9 | 2 | 5 | 2 | 34 |
|  |  | *Conringia orientalis* | 4 | 1 | 5 | 1 | 8 | 3 | 1 | 10 | 33 |
|  |  | *Arabidopsis thaliana* |  |  |  |  | 1 | 2 | 1 | 6 | 10 |
|  |  | *Sinapis alba* | 1 |  | 1 |  | 1 | 1 | 1 | 1 | 6 |
|  |  | *Raphanus* sp. |  |  | 1 |  |  |  | 2 | 1 | 4 |
|  |  | *Rorippa sylvestris* | 1 |  |  |  | 1 |  |  |  | 2 |
|  |  | *Cardamine hirsuta* |  |  | 1 |  |  |  |  |  | 1 |
|  |  | *Diplotaxis tenuifolia* |  |  |  |  | 1 |  |  |  | 1 |
|  |  | ***Sinapis arvensis*** | 1 |  |  |  |  |  |  |  | 1 |
|  |  | *Sisymbrium* sp. |  |  |  |  |  |  |  | 1 | 1 |
| Lamiales | Plantaginaceae | *Plantago major* | 5 | 4 | 4 | 2 | 8 | 1 | 1 | 3 | 28 |
|  | Plantaginaceae | *Plantago lanceolata* | 3 | 3 | 1 |  | 3 |  | 1 | 1 | 12 |
|  | Lamiaceae | ***Galeopsis tetrahit*** |  |  |  | 1 |  |  | 1 |  | 2 |
|  | Lamiaceae | *Salvia* sp. |  | 1 |  |  | 1 |  |  |  | 2 |
|  | Plantaginaceae | ***Veronica arvensis*** |  |  |  |  | 2 |  |  |  | 2 |
|  | Plantaginaceae | ***Veronica persica*** |  |  |  | 1 | 1 |  |  |  | 2 |
|  | Lamiaceae | *Glechoma hederacea* |  |  |  |  |  |  |  | 1 | 1 |
|  | Plantaginaceae | ***Veronica opaca/polita*** |  |  |  |  | 1 |  |  |  | 1 |
| Ranunculales | Ranunculaceae | *Ranunculus repens* | 1 | 3 | 1 |  | 3 |  |  | 4 | 12 |
|  |  | *Ranunculus acris* | 2 |  | 1 |  | 1 | 1 | 1 | 1 | 7 |
|  |  | *Ranunculus bulbosus* |  |  |  |  |  |  |  | 1 | 1 |
| Asparagales | Iridaceae | *Iris* sp. | 1 |  | 2 |  | 2 |  | 1 | 3 | 9 |
| Fabales | Fabaceae | *Trifolium repens* | 2 | 1 |  |  | 1 | 1 | 1 | 3 | 9 |
|  |  | *Trifolium pratense* | 1 | 1 | 1 |  | 1 |  |  |  | 4 |
|  |  | *Medicago sativa* |  |  | 1 |  | 2 |  |  |  | 3 |
|  |  | ***Vicia hirsuta*** |  |  |  |  |  | 1 |  |  | 1 |
|  |  | *Vicia villosa* |  |  | 1 |  |  |  |  |  | 1 |
| Caryophyllales | Chenopodiaceae | *Chenopodium album* | 1 | 3 | 1 | 1 |  |  | 2 |  | 8 |
|  | Chenopodiaceae | *Chenopodium* sp. |  |  |  |  | 3 |  | 1 | 3 | 7 |
|  | Polygonaceae | *Persicaria lapathifolia* | 1 |  |  | 1 | 3 |  |  | 1 | 6 |
|  | Amaranthaceae | *Amaranthus powellii/retroflexus* | 1 |  |  |  |  |  |  |  | 1 |
|  | Caryophyllaceae | *Cerastium fontanum/glomeratum* |  |  |  |  | 1 |  |  |  | 1 |
|  | Caryophyllaceae | *Myosoton aquaticum* |  |  |  |  |  |  | 1 |  | 1 |
|  | Polygonaceae | *Rumex acetosa* |  | 1 |  |  |  |  |  |  | 1 |
|  | Caryophyllaceae | *Stellaria* sp. |  | 1 |  |  |  |  |  |  | 1 |
| Rosales | Urticaceae | *Urtica dioica* | 1 | 3 |  |  | 1 | 2 | 1 |  | 8 |
|  | Rosaceae | *Potentilla reptans* | 1 | 2 |  |  | 2 |  |  |  | 5 |
|  | Cannabaceae | *Cannabis sativa/ruderalis* |  | 1 |  |  |  |  |  |  | 1 |
|  | Rosaceae | *Potentilla* sp. |  | 1 |  |  |  |  |  |  | 1 |
| Solanales | Convolvulaceae | *Convolvulus arvensis* |  |  |  |  | 1 |  | 1 | 3 | 5 |
| Boraginales | Boraginaceae | *Symphytum officinale* | 1 | 1 | 1 |  | 1 |  |  |  | 4 |
|  |  | ***Myosotis arvensis*** | 1 |  |  |  | 1 |  |  |  | 2 |
|  |  | *Borago officinalis* |  |  |  |  |  |  | 1 |  | 1 |
| Myrtales | Onagraceae | *Epilobium ciliatum* | 1 |  |  |  |  |  | 1 | 2 | 4 |
|  | Lythraceae | *Lythrum salicaria* | 1 |  |  |  |  |  |  |  | 1 |
| Apiales | Apiaceae | *Anthriscus sylvestris* | 1 | 1 |  |  | 1 |  |  |  | 3 |
|  |  | *Heracleum sphondylium* |  |  |  |  |  |  |  | 3 | 3 |
|  |  | *Carum carvi* |  |  |  | 1 |  |  | 1 |  | 2 |
|  |  | *Aegopodium podagraria* |  |  |  |  |  |  |  | 1 | 1 |
|  |  | *Aethusa cynapium* |  |  | 1 |  |  |  |  |  | 1 |
| Dipsacales | Caprifoliaceae | *Knautia arvensis* |  |  |  |  | 1 |  |  |  | 1 |
| Ericales | Primulaceae | *Anagallis arvensis* |  |  |  | 1 |  |  |  |  | 1 |
| Gentianales | Rubiaceae | ***Galium aparine*** |  | 1 |  |  |  |  |  |  | 1 |
| **Sum per carabid species** | | | 111 | 98 | 76 | 33 | 131 | 54 | 55 | 142 | 700 |

Table S3: List of the 80 plant species that were amplified in the NGS samples; divided according to the sampling dates; plant species that were included in the seed mixture for treatment 2 and 3 were highlighted in bold.

| **Order** | **Family** | **Plant species** | **Sampling dates** | | | | | **Sum per plant species** |
| --- | --- | --- | --- | --- | --- | --- | --- | --- |
|  |  |  | **S1** | | **S2** | **S3** | **S4** |  |
| Asterales | Asteraceae | *Galinsoga parviflora/quadriradiata* | 7 | | 14 | 46 | 149 | 216 |
| Caryophyllales | Caryophyllaceae | ***Stellaria media*** | 4 | | 15 | 15 | 6 | 40 |
| Poales | Poaceae | *Lolium* sp. | 1 | | 2 | 12 | 24 | 39 |
| Brassicales | Brassicaceae | ***Capsella bursa-pastoris*** | 3 | | 10 | 9 | 12 | 34 |
| Brassicales | Brassicaceae | *Conringia orientalis* | 2 | | 6 | 14 | 11 | 33 |
| Asterales | Asteraceae | *Helianthus annuus* | 1 | | 4 | 15 | 11 | 31 |
| Lamiales | Plantaginaceae | *Plantago major* |  | |  | 3 | 25 | 28 |
| Poales | Poaceae | ***Poa annua/supina*** | 1 | | 7 | 8 | 7 | 23 |
| Poales | Poaceae | *Lolium perenne* | 1 | | 5 | 4 | 11 | 21 |
| Asterales | Asteraceae | ***Taraxacum officinale*** | 1 | | 3 | 5 | 8 | 17 |
| Poales | Poaceae | *Dactylis glomerata* |  | | 2 | 3 | 8 | 13 |
| Lamiales | Plantaginaceae | *Plantago lanceolata* | 3 | | 3 | 4 | 2 | 12 |
| Ranunculales | Ranunculaceae | *Ranunculus repens* |  | | 2 | 3 | 7 | 12 |
| Brassicales | Brassicaceae | *Arabidopsis thaliana* |  | | 2 | 2 | 6 | 10 |
| Asparagales | Iridaceae | *Iris* sp. |  | | 4 |  | 5 | 9 |
| Fabales | Fabaceae | *Trifolium repens* | 1 | |  | 4 | 4 | 9 |
| Caryophyllales | Chenopodiaceae | *Chenopodium album* | 1 | |  | 3 | 4 | 8 |
| Rosales | Urticaceae | *Urtica dioica* |  | | 1 |  | 7 | 8 |
| Caryophyllales | Chenopodiaceae | *Chenopodium* sp. | 1 | | 2 | 3 | 1 | 7 |
| Ranunculales | Ranunculaceae | *Ranunculus acris* | 1 | | 2 | 1 | 3 | 7 |
| Caryophyllales | Polygonaceae | *Persicaria lapathifolia* |  | |  | 3 | 3 | 6 |
| Poales | Poaceae | *Phleum pratense* | 1 | |  | 2 | 3 | 6 |
| Brassicales | Brassicaceae | *Sinapis alba* | 3 | |  | 3 |  | 6 |
| Solanales | Convolvulaceae | *Convolvulus arvensis* | 3 | | 2 |  |  | 5 |
| Rosales | Rosaceae | *Potentilla reptans* |  | | 1 | 2 | 2 | 5 |
| Poales | Poaceae | *Alopecurus pratensis* |  | | 1 | 1 | 2 | 4 |
| Myrtales | Onagraceae | *Epilobium ciliatum* |  | | 1 | 2 | 1 | 4 |
| Brassicales | Brassicaceae | *Raphanus* sp. | 3 | |  |  | 1 | 4 |
| Boraginales | Boraginaceae | *Symphytum officinale* |  | |  |  | 4 | 4 |
| Fabales | Fabaceae | *Trifolium pratense* |  | |  | 3 | 1 | 4 |
| Poales | Poaceae | *Trisetum flavescens* |  | |  | 4 |  | 4 |
| Asterales | Asteraceae | *Achillea millefolium* | 1 | |  | 1 | 1 | 3 |
| Apiales | Apiaceae | *Anthriscus sylvestris* |  | |  |  | 3 | 3 |
| Apiales | Apiaceae | *Heracleum sphondylium* | 1 | |  |  | 2 | 3 |
| Asterales | Asteraceae | *Leontodon hispidus* |  | |  | 2 | 1 | 3 |
| Fabales | Fabaceae | *Medicago sativa* | 1 | |  |  | 2 | 3 |
| Poales | Poaceae | *Poa trivialis* |  | | 1 |  | 2 | 3 |
| Poales | Poaceae | ***Apera spica-venti*** | 1 | | 1 |  |  | 2 |
| Poales | Poaceae | *Arrhenatherum elatius* | 1 | | 1 |  |  | 2 |
| Apiales | Apiaceae | *Carum carvi* |  | |  |  | 2 | 2 |
| Lamiales | Lamiaceae | ***Galeopsis tetrahit*** |  | | 1 |  | 1 | 2 |
| Asterales | Asteraceae | *Matricaria chamomilla* |  | |  | 2 |  | 2 |
| Boraginales | Boraginaceae | ***Myosotis arvensis*** |  | |  |  | 2 | 2 |
| Brassicales | Brassicaceae | *Rorippa sylvestris* |  | |  |  | 2 | 2 |
| Lamiales | Lamiaceae | *Salvia* sp. |  | | 2 |  |  | 2 |
| Lamiales | Plantaginaceae | ***Veronica arvensis*** |  | |  | 1 | 1 | 2 |
| Lamiales | Plantaginaceae | ***Veronica persica*** |  | |  | 1 | 1 | 2 |
| Apiales | Apiaceae | *Aegopodium podagraria* |  | |  |  | 1 | 1 |
| Apiales | Apiaceae | *Aethusa cynapium* |  | |  | 1 |  | 1 |
| Poales | Poaceae | *Agrostis* sp. | 1 | |  |  |  | 1 |
| Caryophyllales | Amaranthaceae | *Amaranthus powellii/retroflexus* |  | |  |  | 1 | 1 |
| Ericales | Primulaceae | *Anagallis arvensis* | 1 | |  |  |  | 1 |
| Asterales | Asteraceae | *Artemisia vulgaris* |  | |  | 1 |  | 1 |
| Asterales | Asteraceae | *Bellis perennis* |  | |  | 1 |  | 1 |
| Boraginales | Boraginaceae | *Borago officinalis* |  | |  | 1 |  | 1 |
| Rosales | Cannabaceae | *Cannabis sativa/ruderalis* |  | |  | 1 |  | 1 |
| Brassicales | Brassicaceae | *Cardamine hirsuta* |  | | 1 |  |  | 1 |
| Caryophyllales | Caryophyllaceae | *Cerastium fontanum/glomeratum* |  | |  | 1 |  | 1 |
| Asterales | Asteraceae | *Cirsium* sp. |  | |  |  | 1 | 1 |
| Asterales | Asteraceae | *Crepis* sp. |  | |  |  | 1 | 1 |
| Brassicales | Brassicaceae | *Diplotaxis tenuifolia* |  | | 1 |  |  | 1 |
| Asterales | Asteraceae | *Erigeron canadensis* |  | |  | 1 |  | 1 |
| Poales | Poaceae | *Festuca pratensis* |  | |  | 1 |  | 1 |
| Poales | Poaceae | *Festuca rubra* |  | |  |  | 1 | 1 |
| Gentianales | Rubiaceae | ***Galium aparine*** |  | |  | 1 |  | 1 |
| Lamiales | Lamiaceae | *Glechoma hederacea* |  | |  | 1 |  | 1 |
| Dipsacales | Caprifoliaceae | *Knautia arvensis* |  | |  | 1 |  | 1 |
| Myrtales | Lythraceae | *Lythrum salicaria* |  | |  | 1 |  | 1 |
| Caryophyllales | Caryophyllaceae | *Myosoton aquaticum* |  | |  |  | 1 | 1 |
| Poales | Poaceae | *Poa pratensis* | 1 | |  |  |  | 1 |
| Rosales | Rosaceae | *Potentilla* sp. |  | |  | 1 |  | 1 |
| Ranunculales | Ranunculaceae | *Ranunculus bulbosus* | 1 | |  |  |  | 1 |
| Caryophyllales | Polygonaceae | *Rumex acetosa* |  | | 1 |  |  | 1 |
| Brassicales | Brassicaceae | ***Sinapis arvensis*** |  | |  |  | 1 | 1 |
| Brassicales | Brassicaceae | *Sisymbrium* sp. |  | |  |  | 1 | 1 |
| Caryophyllales | Caryophyllaceae | *Stellaria* sp. |  | | 1 |  |  | 1 |
| Asterales | Asteraceae | ***Tripleurospermum inodorum*** |  | |  | 1 |  | 1 |
| Lamiales | Plantaginaceae | ***Veronica opaca/polita*** |  | |  |  | 1 | 1 |
| Fabales | Fabaceae | ***Vicia hirsuta*** |  | |  |  | 1 | 1 |
| Fabales | Fabaceae | *Vicia villosa* |  | |  | 1 |  | 1 |
| **Sum per sampling date** | | | | 47 | 99 | 196 | 358 | 700 |

**Supplementary figures**


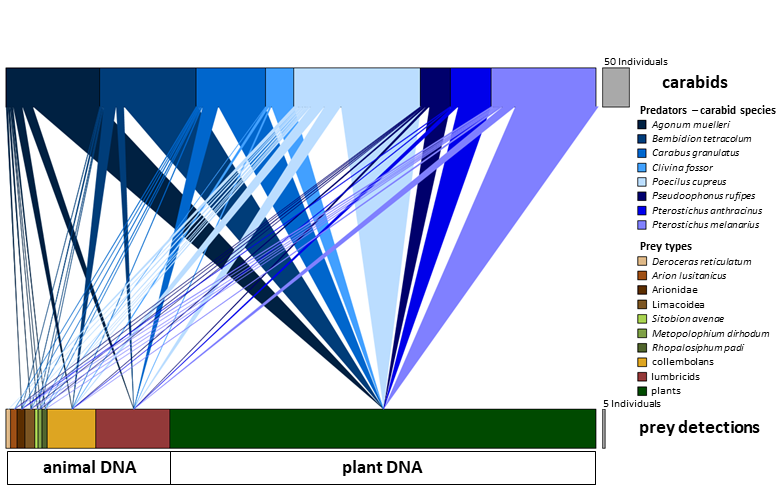


Figure S1: Food web of carabid species in Rotholz 2017. The prey detections indicate in how many samples the respective prey could be positively amplified by PCR. The vertical bars show in which carabid species they were found.


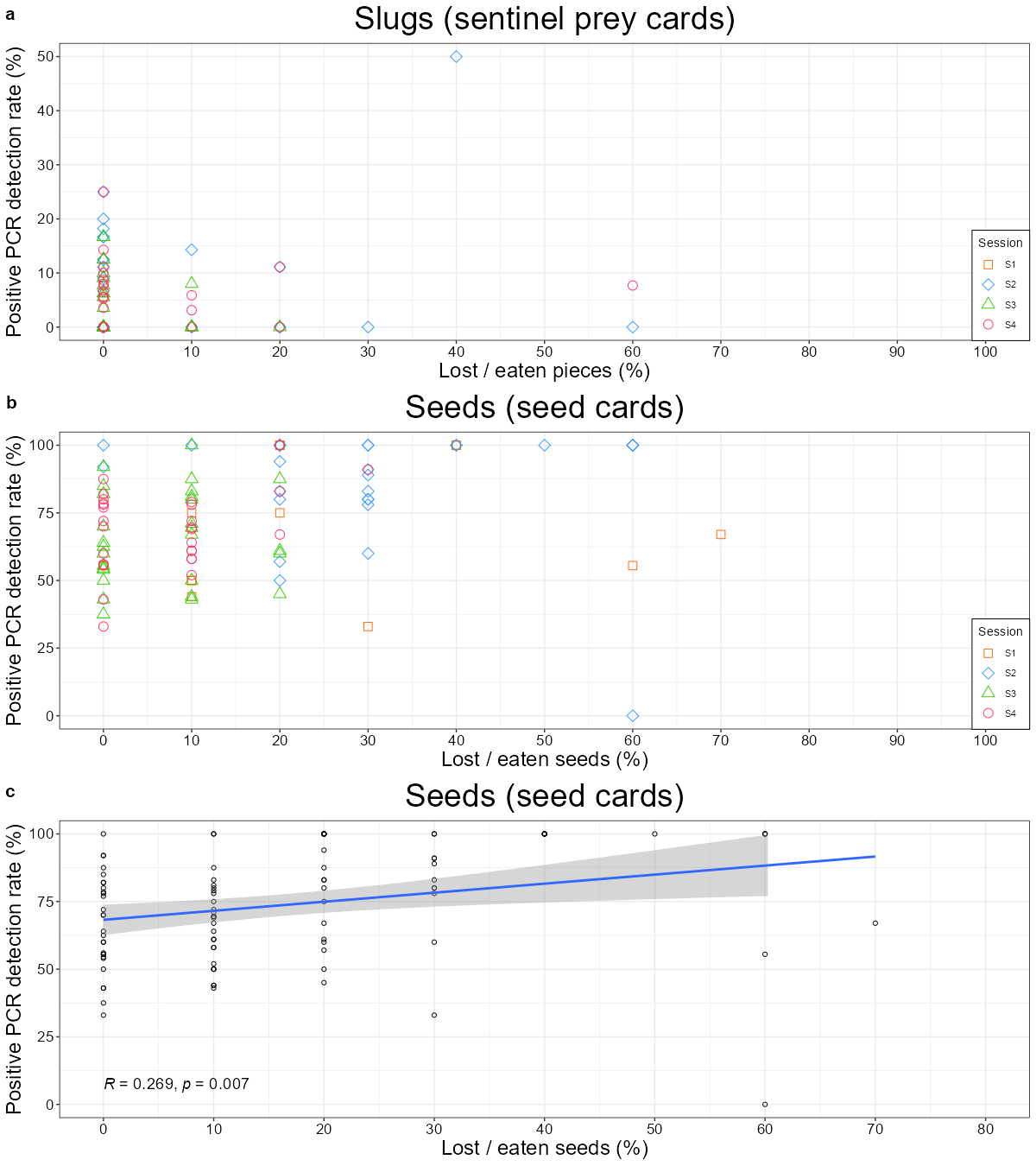


Figure S2: Comparison of the individual positive PCR detection rates for (a) slugs and (b) plants with the percentage of lost or eaten pieces or seeds of the corresponding sentinel card of the respective plot and session. A linear relationship for the data could only be obtained for the plants/seeds and was shown in (c) with the corresponding Pearson correlation and the associated 95% confidence interval (shaded).


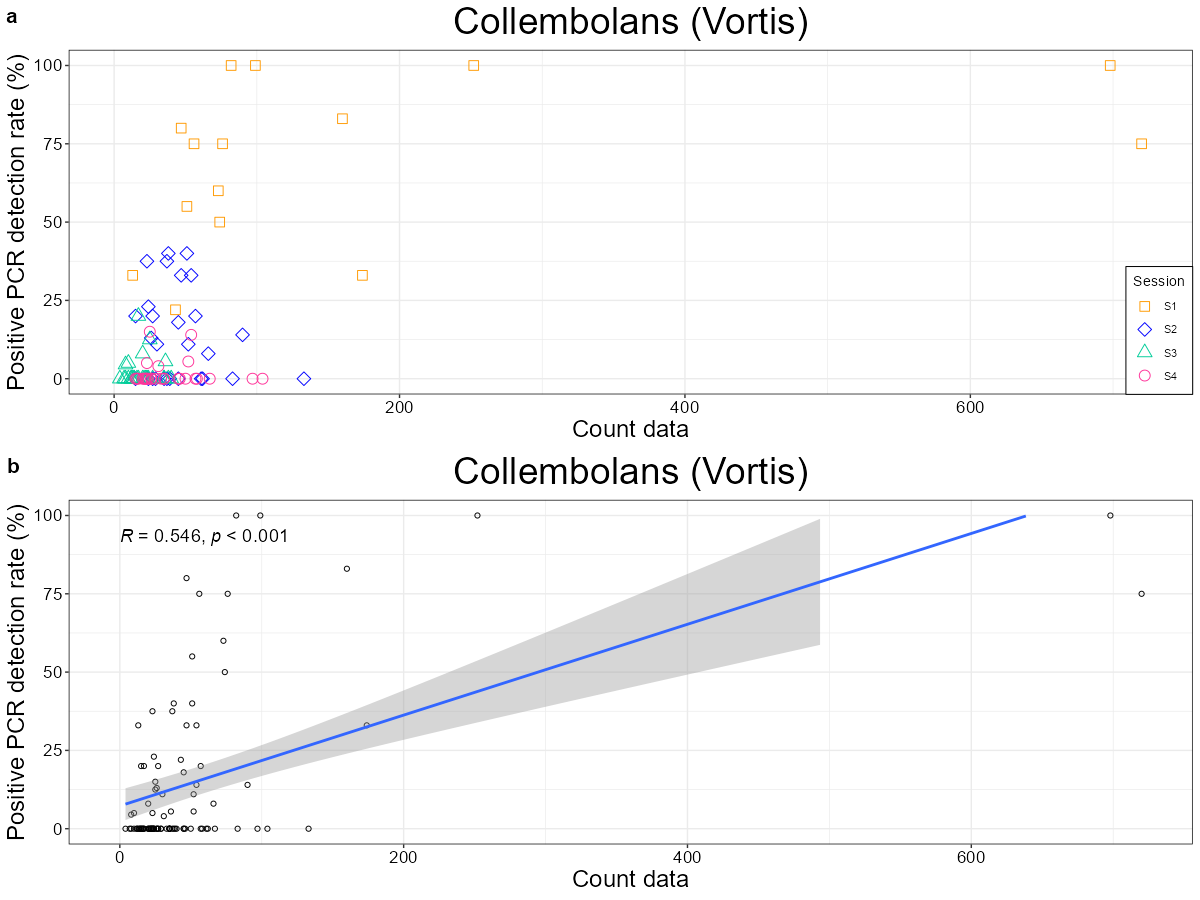


Figure S3: Comparison of the individual positive PCR detection rates for collembolans with the count data of these in Vortis samples: (a) divided into plot and session and (b) with the corresponding Pearson correlation to the data and the associated 95% confidence interval (shaded).
